# A Review of the Phytochemistry and Pharmacology of *Phyllanthus urinaria* L.

**DOI:** 10.3389/fphar.2018.01109

**Published:** 2018-10-01

**Authors:** Madamanchi Geethangili, Shih-Torng Ding

**Affiliations:** Department of Animal Science and Technology, National Taiwan University, Taipei, Taiwan

**Keywords:** *Phyllanthus urinaria*, crude extracts, phytochemical constituents, biological activities, clinical trials

## Abstract

The genus *Phyllanthus* (L.) is one of the most important groups of plants belonging to the Phyllantaceae family. *Phyllanthus urinaria* (L.) is an annual perennial herbal species found in tropical Asia, America, China, and the Indian Ocean islands. *P. urinaria* is used in folk medicine as a cure to treat jaundice, diabetes, malaria, and liver diseases. This review provides traditional knowledge, phytochemistry, and biological activities of *P. urinaria*. The literature reviewed for this article was obtained from the Web of Science, SciFinder, PubMed, ScienceDirect, and Google Scholar journal papers published prior to December 2017. Phytochemical investigations reveal that the plant is a rich source of lignans, tannins, flavonoids, phenolics, terpenoids, and other secondary metabolites. Pharmacological activities include anticancer, hepatoprotective, antidiabetic, antimicrobial, and cardioprotective effects. Thus, this present review summarizes the phytochemical constituents and their biological activities including biological studies on various crude extracts and fractions both *in vitro* and *in vivo*, and on clinical trial information about *P. urinaria*. This review compiles 93 naturally occurring compounds from *P. urinaria* along with their structures and pharmacological activities. The review is expected to stimulate further research on *P. urinaria*, and its pharmacological potential to yield novel therapeutic agents.

## Introduction

Traditional or indigenous medicine denotes medical practices developed by local ethnic people using natural herbs. Different world locations have their own history of traditional medicine. For example, Ayurveda medicine originated from Southeast Asia, Unani medicine originated from Arab countries in the Middle East, and acupuncture and traditional Chinese medicine (TCM) originated from China (Tao et al., 2014). Traditionally herbal medicines are used in folk medicine for the treatment of various health complications including inflammatory, cancerous, diabetic, hypertensive, and cardiovascular diseases (Tao et al., 2014). Medicinal plants are rich sources for new drug discovery as evidenced by some recent drugs that are from plant-derived compounds/derivatives (Harvey et al., 2015). For example, success using classic traditional medicine includes salicylic acid and artemisinin, possibly the most effective medicinal natural products ever found. The use of traditional medicinal data in the drug discovery process results in new therapeutics, and identifies leads that undergo clinical trials (Harvey et al., 2015). In general, it is believed that traditional medicines are safe and harmless as compared with modern drugs although this is seldom rigorously tested. Indications that the natural product extracts are effective against a particular pathological condition are based on the literature and do not imply that the effect has been proven using double blind studies with placebos (Izzo et al., 2016). The modern approach has the goal to establish evidence-based use of traditional medicines, both locally and globally.

### Phyllanthus urinaria

The genus *Phyllanthus* (L.) belongs to a family of flowering plants Phyllanthaceae and consists of more than 1000 species widely distributed in various parts of the world (Mao et al., 2016). The species of this genus including trees, herbs and shrubs that are pharmacologically valuable as they contain various bioactive compounds (Calixto et al., 1998; Mao et al., 2016). Previous scientific data indicate that more than 500 chemical compounds (phytochemicals) have been isolated from species of the genus *Phyllanthus* (Mao et al., 2016). It is interesting to note that crude extracts obtained from species of *Phyllanthus* have inhibitory effects on the hepatitis B virus (HBV). Previous reviews broadly highlight the biological activities of *Phyllanthus* species, mostly from *P. amurus* Schum. & Thonn.*, P. emblica* L. or *P. niruri* L. (Calixto et al., 1998; Mao et al., 2016; Kaur et al., 2017; Tewari et al., 2017; Yadav et al., 2017). However, there is no specific and detailed review of *P. urinaria*. To provide scientific proof for *P. urinaria* ethnopharmacological and traditional uses, recent scientific studies focus on its chemical constituents and their biological properties. Therefore, this review provides information about *P. urinaria* including comprehensive information about the traditional use of *P. urinaria*, its phytochemicals and their biological activities. It also includes biological studies both *in vitro* and *in vivo* on various extracts of *P. urinaria*, analysis of pure compounds and clinical trial information.

## Methodology

The literature for this review was collected from various search engines and databases including Scifinder, Web of Science, PubMed, Google Scholar, and ScienceDirect. We considered the literature published prior to December 2017 on ethnopharmacological uses, pharmacology of extracts, and isolated pure compounds from different parts of *P. urinaria*. The search terms “*Phyllanthus urinaria*,” or “*P. urinaria* extract,” or “*P. urinaria* compound” were used with no exact time limit. Potential full-texts of eligible papers were identified. All articles with title/abstract were included and no language restrictions applied. All relevant references were checked for additional and unpublished citations.

## Medicinal uses of *P. urinaria*: traditional knowledge

Traditionally, the whole plant, roots, fruits, and leaves of *P. urinaria* is used for the treatment of various complications in different regions of the world. In particular, the Chinese and Indian traditional medicine system documents different applications of parts of this plant as remedies for various health complications. For example, in traditional Chinese medicine (TCM), decoction of the whole plant of *P. urinaria* (Chinese name: Yexiazhu) can clear heat-toxin and remove dampness so it is employed to treat jaundice, enteritis, diarrhea, and dropsy (Xia, 1997). The TCM prescription, named “yexiazhu capsule,” claims to cure hepatitis B (Xia, 1997). In India, *P. urinaria* is considered a very good diuretic, and the crushed plant is used as a fish poison (Bharali et al., 2003). In Taiwan, decoction of young shoots or roots of *P. urinaria* is traditionally used to treat contagious hepatitis, acute conjunctivitis, diarrhea, edema, dysentery etc. (Lee et al., 2006). In Thailand, *P. amarus, P. virgatus* G. Forst., and *P. urinaria* share the name “look tai bai”; all of these plants are used to treat gonorrhea, jaundice, diabetes, and liver disease (Suthienkul et al., 1993; Chudapongse et al., 2010). In Malaysia, the juice is applied to stimulate children's appetite and to wash their tongues (Jantan et al., 2014). In Papua New Guinea, an extract is used as a febrifuge. In Brunei, a leaf poultice is applied with coconut milk to treat smallpox. In Cambodia, *P. urinaria* is used against malaria. The pills prepared from equal amounts of *P. urinaria* leaves and black pepper are beneficial for malarial fever (Hout et al., 2006). In Ghana, a decoction is employed to treat dysentery and in the Solomon Islands, the leaves are used to relieve pain in the chest (Agyare et al., 2014). In Madagascar, stem or leaf infusions are used to treat bronchitis and asthma (Calixto et al., 1998). In South America, a decoction is used for the treatment of kidney stones (Hout et al., 2006). Besides conventional usage, modern day scientific investigations have now confirmed pharmacological properties of *P. urinaria*. These previous studies suggest that *P. urinaria* is an effective medicinal remedy to treat and prevent a wide range of disorders.

## Pharmacological activities of *P. urinaria* extracts

### Anticancer activities

Epidemiological and experimental studies suggest that medicinal herbs have great potential in the management of different types of cancers including lung, breast, colon, liver, prostate, skin, and ovarian carcinomas. In this connection, medicinal plant extracts, and their purified compounds (phytochemicals) have significant growth inhibitory potential against various types of cancer cells *in vitro* as well as *in vivo* (Harvey et al., 2015). Although *P. urinaria* preparations traditionally are used as an alternative medicine for various cancers, there is little scientific evidence available about the use of *P. urinaria* as an anticancer agent (Table 1). Reported scientific data indicate that the anticancer signaling mechanism of *P. urinaria* extracts is through induction of apoptosis. Table 1 summarizes the inhibitory potential of *P. urinaria* extracts against various types of cancer cells. An aqueous extract obtained from the whole *P. urinaria* plant has growth inhibitory activity in different types of cancer cells including hepatoma, leukemia, and fibrosarcoma through induction of apoptosis; normal endothelial cell lines and liver cells are not affected (Huang et al., 2003, 2004a,b). The aqueous extract reduces proliferation of Lewis lung carcinoma cells and human myeloid leukemia cells (HL-60 cells) in a dose- and time-dependent manner, without affecting the normal cells (Huang et al., 2003). Growth inhibition of HL-60 cells is associated with induction of the apoptosis signaling pathway and Fas receptor/ligand expression in CD95 cells (Huang et al., 2004b). Additionally, aqueous extracts of *P. urinaria* affected the human umbilical cord endothelial cells (HUVEC) by reduced blood vessel density, matrix induced tube formation, and cell migration (Huang et al., 2006). The aqueous and methanolic extracts obtained from the whole plant of *P. urinaria* inhibits metastasis of breast carcinoma cells (MCF-7) through extracellular signal-related kinase (ERK) and hypoxia pathways (Lee et al., 2011, 2016). An aqueous extract obtained from the whole *P. urinaria* plant cause cytotoxic effects in various types of cancer cells by induction of DNA fragmentation and cell apoptosis along with increased caspase-3 activity and reduced telomerase activity (Huang et al., 2009, 2010). It is reported that both aqueous and methanolic extracts of *P. urinaria* whole plant inhibit proliferation, metastasis and angiogenesis in a human melanoma (MeWo) cancer cell line through MAPKs, Myc/Max, NFκB, and hypoxia pathways (Tang et al., 2010, 2014). Both aqueous and methanolic extracts of *P. urinaria* whole plant inhibit A549 cell metastasis by suppressed invasion and migration of A549 cells through the ERK1/2 and hypoxia signaling pathways (Lee et al., 2013b). The hot water extract from whole plants of *P. urinaria* induces apoptosis in human osteosarcoma 143B cells through the Fas receptor/ligand expression pathway (Wu et al., 2012). The same extract inhibits invasion and migration of another osteosarcoma cell line, Saos-2 cells through the ERK and Akt signaling pathways (Lu et al., 2013). Methanol extracts of *P. urinaria* aerial parts has anti-angiogenic properties against rat aortic vascular growth (Ng et al., 2010). The matrix metalloproteinases (MMPs) promote the prevention of metastasis of cancer cells. *P. urinaria* extracts inhibit the invasion and migration of highly metastatic A549 and Lewis lung carcinoma (LLC) cells through decreased expression of matrix MMP-2 and MMP-9, as well as transcription of MMP-2 mRNA, suggesting suppression of the function of MMPs by extracts [ethanol/water (1:1)] obtained from *P. urinaria* leaves (Tseng et al., 2012). These effects may relate to the presence of several cytotoxic and anticancer compounds in *P. urinaria* extracts. Therefore, further studies require identification of the responsible compounds for the observed anticancer activity. The results of the above studies validate the traditional claim of the anticancer activity of *P. urinaria*, and thus it might serve as a potential source of potent anticancer agents.

**Table 1:** Reported biological activities *in vitro* and *in vivo* of *Phyllanthus urinaria* crude extracts and fractions.

**Extract**	**Reported activity**	**References**
95% ethanolic extract from whole plant	Chondroprotective	Buddhachat et al., 2017
Aqueous extract from whole plant	Hepatoprotection against CCl_4_-induced liver injury	Guo et al., 2017
Fractions of acetone extract from whole plant	anti-HCV	Chung et al., 2016
Aqueous extract from dried leafs	Inhibit lamivudine resistant hepatitis B virus	Jung et al., 2015
Aqueous and methanolic extract from whole plant	Anticancer against MCF-7 metastasis	Lee et al., 2016
Ethanolic extract from whole plant	α-glucosidase inhibition	Trinh et al., 2016
Aqueous extract from commercial plant	Inhibits hepatitis B virus replication and expression in hepatitis B virus transfection model *in vitro*	Wu et al., 2015
Methanol extract from whole plant	Antiplasmodial activity	Haslinda et al., 2015
Fractions from whole plant methanol extract	Antiviral activity against Human enterovirus 71 (EV71) and Coxsackievirus A16 (CA16) infections.	Yeo et al., 2015
Aqueous extract from whole plant	Antiviral activity against duck hepatitis B virus *in vitro*	Chen et al., 1995
Ethanol/water (50:50 v/v) extract from whole plant	Anthelmintic against free-living nematode *Caenorhabditis elegans*	Agyare et al., 2014
Aqueous and methanolic extracts from whole plant	Inhibited proliferation, metastasis and angiogenesis in human melanoma (MeWo) cancer cell line through MAPKs, Myc/Max, NFκB, and hypoxia pathways	Tang et al., 2014
Aqueous and methanolic extracts from whole plant	Inhibited metastasis in human lung (A549) cancer cell line through Raf-MEK-ERK and Hypoxia pathways	Lee et al., 2013b
Cocktail extract from whole plant	Inhibited dengue virus 2	Lee et al., 2013a
Ethanol/water (50:50 v/v) extract from whole plant	Suppressed human osteosarcoma Saos-2 cell invasion and migration by transcriptionally inhibiting u-PA via ERK and Akt signaling pathways	Lu et al., 2013
Aqueous extract from whole plant	Antiviral against herpes simplex virus type-1 (HSV-1) and HSV-2 in Vero cells	Tan et al., 2013
Aqueous and methanolic extract from whole plant	Suppressed prostate cancer cell line PC-3 cells proliferation and induced apoptosis through MAPKs, PI3K/Akt, NFκB, and Hypoxia pathways	Tang et al., 2013
Methanolic extract from whole plant	Inhibited phagocytic activity of human neutrophils	Yuandani et al., 2013
Ethanol/water (50:50 v/v) extract from leaves	Antimetastatic potentials against A549 cells	Tseng et al., 2012
Methanol/water (50:50 v/v) extract from leaves	Mild inhibitory activity against porcine pancreatic amylase	Gunawan-Puteri et al., 2012
Aqueous extract from whole plant	Anti-angiogenic	Huang et al., 2011
Aqueous and methanolic extract from whole plant	Antimetastatic in human lung (A549) and Breast (MCF-7) cancer cell lines	Lee et al., 2011
Methanolic extract from whole plant	Hepatoprotective activity against tert-butyl hydroxide (t-BH)-induced cytotoxicity in HepG2 cell line	Sharma et al., 2011
Methanol/water (50:50 v/v) extract from whole plant	Induced cell death of HepG2 cells	Chudapongse et al., 2010
Methanolic extract from aerial parts	anti-angiogenic against rat aortic vascular growth	Ng et al., 2010
Ethanolic extract from whole plant	Oral administration of *P. urinaria* extract attenuated the acetaminophen induced hepatotoxicity, and inhibition of cytochrome P450 CYP2E1 enzyme in mice	Hau et al., 2009
Ethanolic extract from whole plant	Protected cardiac H9c2 cells against doxorubicin-induced by influencing the nuclear localization of glutathione-S transferase Pi without affecting enzymatic activity.	Chularojmontri et al., 2009
Chloroform and methanol extracts from whole plant	Inhibited *Helicobacter pylori*, and its adhesion and invasion to AGS cells	Lai et al., 2008
480 mg Korean *P. urinaria* extract capsule	Alleviated the MCD-induced nutritional steatohepatitis through reduced oxidative stress, inflammation, and lipid accumulation	Shen et al., 2008
*Phyllanthus urinaria* extract	*In vivo* promote the N-cadherin expression in the testis tissues disrupted by nitrogen mustard (HN2)	Zhang et al., 2008
Aqueous extract from whole plant	*In vitro* antiplasmodial activity	Hout et al., 2006
Methanolic extract from whole plant	*In vivo* hepatoprotection against CCl_4_-induced liver damage	Lee et al., 2006
Ethanolic extract from aerial part	Antioxidative and Cardioprotective	Chularojmontri et al., 2005
Acetone, ethanol and methanol extracts from whole plant	Inhibited HSV-2 but not HSV-1 infection	Yang et al., 2005
Aqueous extract from whole plant	*In vitro* growth cell inhibition in hepatoma, leukemia, fibrosarcoma and HUVEC cells	Huang et al., 2003, 2004a,b, 2006
A fraction containing 60% corilagin	*In vivo* antithrombosis due to its inhibition of platelet-neutrophil adhesion.	Shen et al., 2004
Hydro-alcoholic extract from whole plant	Chemopreventive property against 7,12-dimethylbenz-anthracene (DMBA)-induced skin papillomagenesis in mice.	Bharali et al., 2003
Hydro-alcoholic extract of stems, leaves and roots	Caused a graded relaxation in guinea-pig trachea (GPT) pre-contracted by carbachol.	Paulino et al., 1996a
Hydro-alcoholic extract of stems, leaves and roots	Caused graded contraction in GPT modulated by the epithelium, depends on the release of a cyclo-oxygenase metabolite, and relies largely upon an extracellular Ca^2+^ influx	Paulino et al., 1996b
Hydro-alcoholic extract of stems, leaves and roots	Antinociceptive effect in mice	Santos et al., 1995
Hydroalcoholic extract substance P and substance P methyl ester	Caused graded contractions in the guinea-pig urinary bladder	Dias et al., 1995
50% methanolic extract from whole plant	Oral administration (30 mg/kg) decreased the blood glucose levels	Higashino et al., 1992

The majority of previous scientific reports focus on growth inhibitory potential of *P. urinaria* extracts in various cancer cell lines *in vitro*. An aqueous *P. urinaria* whole plant extract has anti-angiogenesis and reduced tumor growth in Lewis lung carcinoma *in vivo* (Huang et al., 2003, 2006). Oral administration of an aqueous *P. urinaria* extract inhibits human osteosarcoma xenograft growth in mice through modulation of the mitochondrial fission/fusion machinery (Huang et al., 2014, 2016). Although the cytotoxic and anticancer activities of *P. urinaria* extracts seem promising from the reported studies, the lack of toxicity studies with appropriate normal cells, and lack of comparison with positive control drugs further restricts the current knowledge on *P. urinaria* as an anticancer agent.

### Hepatoprotective and antioxidant action of *P. urinaria*

Liver damage can be caused by hepatitis virus infection, poor eating habits, heavy metal intoxication, alcohol intake or obstruction of the biliary tract (Zhong et al., 2013). Recent studies indicate that naturally derived products have significant hepatoprotective properties through their antioxidant, anti-inflammatory and anticancer properties (Ali et al., 2018). Chronic hepatitis B is a major problem of worldwide concern (Tang et al., 2018). The traditional use of *P. urinaria* as therapy for virus caused-hepatitis suggests that this plant species is an antiviral agent (Ji et al., 1993; Wang et al., 1994; Zhou et al., 1997; Peng et al., 2006; Liu et al., 2008). Previous scientific data also indicate that *P. urinaria* has potential for the treatment of liver diseases (Tables 1, 2). For example, the methanol, acetone and ethanol extracts of *P. urinaria* inhibit Herpes simplex virus (HSV)-2 infection *in vitro* (Yang et al., 2005). Methanolic extracts of *P. urinaria* whole plant inhibit CCl_4_-induced acute liver damage through modulation of serum glutamate-pyruvate-transaminase and glutathione peroxidase *in vivo* (Prakash et al., 1995; Lee et al., 2006). These results are supported by a recent study *in vivo* indicating that *P. urinaria* attenuates CCl_4_-induced hepatotoxicity by regulation of L-carnitine, taurocholic acid, and amino acid metabolisms (Guo et al., 2017). Acetone extracts from whole plant of *P. urinaria* inhibit Hepatitis C virus infection *in vitro* (Chung et al., 2016). An aqueous extract from dried leaves of *P. urinaria* inhibits HBsAg, and HBcAg secretion and Hepatitis B virus (HBV) DNA synthesis in HBV wild type and LMV-resistant-infected HepG2 cells via the COX-2 and IL-6 signaling pathways (Jung et al., 2015). An aqueous *P. urinaria* extract inhibits HBV replication and expression in a HBV transient transfection model *in vitro* (Wu et al., 2015). Sharma et al. (2011) reported that a methanolic extract of *P. urinaria* whole plant protects the Hep G2 cell line against tert-butyl hydroxide (t-BH)-induced cytotoxicity. An ethanolic extract of *P. urinaria* whole plant attenuates the acetaminophen-induced hepatotoxicity and inhibition of the cytochrome P450 CYP2E1 enzyme in mice (Hau et al., 2009). A *P. urinaria* extract (480 mg capsule), contains corilagin, flavonoids and polysaccharides; it attenuates steatohepatitis in cultured hepatocytes *in vitro* and in methionine-and-choline-deficient diet–fed mice *in vivo* (Shen et al., 2008). *P. urinaria* has anti-steatohepatitis effects through its anti-inflammatory activity (reduced TNF-α and IL-6 production through JNK and NF-κB pathways), induction of fatty acid oxidation (upregulation of CYP4a10 and suppression of C/EBPβ), and antioxidant properties (reduced CYP2e1 expression) (Shen et al., 2008). Xu et al. (2007) indicate that a 60% aqueous acetone extract from the whole *P. urinaria* plant has antioxidant activity in the 1,1-diphenyl-2-picrydydrazyl (DPPH)-radical assay with an SC_50_ (50%-scavenging concentrations) value of 14.3 mg/mL). The presence of flavonoids, tannins and phenolic compounds in *P. urinaria* suggest that they contribute the observed antioxidant activity. *P. urinaria* extracts have anti-nociceptive effects in mice (Santos et al., 1995, 1999), liver cell protection against CCl_4_-injury (Zhou et al., 1997), relaxation of guinea pig trachea (Paulino et al., 1996a,b) and induction of the contractile response in urinary bladder (Dias et al., 1995). The aqueous extract from whole plant of *P. urinaria* inhibit HBV DNA polymerase inhibition *in vitro* (Chen et al., 1995). The cocktail extract from whole plant of *P. urinaria* help to reduce activity of dengue virus-2 (Lee et al., 2013a). The ethyl acetate and *n*-butanol fractions from a MeOH extract of *P. urinaria* exhibit antiviral activity against enterovirus 71 (EV71), coxsackie virus A16, and CA16 (Yeo et al., 2015). Aqueous extracts of *P. urinaria* whole plant have antiviral activity against herpes simplex virus type-1 (HSV-1) and HSV-2 with selective index (SI) value >33.6 [(SI = 50% cytotoxic concentration (CC_50_)/ half inhibitory concentration (IC_50_)]; the *P. urinaria* extract may act against the early infection stage and the replication stage in cells *in vitro* (Tan et al., 2013). The ability of *P. urinaria* to inhibit the replication of HBV *in vivo* and *in vitro* indicates its consideration as a potential therapeutic for HBV infection.

**Table 2:** Isolated pure compounds from *Phyllanthus urinaria* and their biological activities.

**No**.	**Compound name**	**Reference for isolation**	**Reported activity**	**Reference for activity**
**LIGNANS**				
**1**	Phyllanthin	Chang et al., 2003; Fang et al., 2008	Antioxidant, antiinflammatory and anticancer	Fang et al., 2008
			Anti *H. pylori*	Lai et al., 2008
			Modulate the vascular tension	Inchoo et al., 2011
			Immunomodulatory	Jantan et al., 2014
			Hepatoprotective	Krithika et al., 2009
**2**	5-Demethoxyniranthin	Chang et al., 2003		
**3**	Niranthin	Chang et al., 2003; Thanh et al., 2014		
**4**	Phyltetralin	Chang et al., 2003; Fang et al., 2008	Antioxidant, antiinflammatory and anticancer	Fang et al., 2008
			Anti *H. pylori*	Lai et al., 2008
**5**	Hypophyllanthin	Chang et al., 2003; Thanh et al., 2014	Modulate the vascular tension	Inchoo et al., 2011
			Cytotoxic to CHO and J774 cells	Thanh et al., 2014
			Immunomodulatory	Jantan et al., 2014
**6**	Nirtetralin	Chang et al., 2003		
**7**	Urinatetralin	Chang et al., 2003		
**8**	Lintetralin	Chang et al., 2003		
**9**	Isolintetralin	Chang et al., 2003		
**10**	Heliobuphthalmin lactone	Chang et al., 2003; Thanh et al., 2014	Cytotoxic to CHO and J774 cells	Thanh et al., 2014
**11**	Dextrobursehernin	Chang et al., 2003		
**12**	Urinaligran	Chang et al., 2003		
**13**	Virgatusin	Chang et al., 2003		
**14**	(+)-Dihydrocubebin	Hu et al., 2014		
**15**	(+)-Lyoniresiol	Hu et al., 2014		
**16**	(7R,7′R,8S,8′S)-Icariol A2	Hu et al., 2014		
**17**	4-Oxopinoresinol	Hu et al., 2014		
**18**	(-)-Syringaresinol	Hu et al., 2014		
**19**	(-)-Episyringaresinol	Hu et al., 2014		
**20**	Evofolin B	Hu et al., 2014		
**21**	Neonirtetralin or Nirtetralin A	Thanh et al., 2014	Cytotoxic to CHO and J774 cells	Thanh et al., 2014
**22**	7′-hydroxy-3′,4′,5,9,9′-pentamethoxy-3,4-methylenedioxy lignin	Giridharan et al., 2002	Anticancer	Giridharan et al., 2002
**TANNINS**				
**23**	Repandinin B	Xu et al., 2007	Antioxidant	Xu et al., 2007
**24**	Repandinin A	Xu et al., 2007	Antioxidant	Xu et al., 2007
**25**	Furosin	Xu et al., 2007	Antioxidant	Xu et al., 2007
**26**	Geraniin	Zhang et al., 2000b; Xu et al., 2007; Wu et al., 2012	Anticancer	Zhai et al., 2016
			Antioxidant	Xu et al., 2007
			Immunomodulatory	Jantan et al., 2014
			Antioxidant and antihypertensive	Lin et al., 2008
**27**	Repandusinic acid A	Xu et al., 2007; Trinh et al., 2016	α-glucosidase inhibition	Trinh et al., 2016
			Antioxidant	Xu et al., 2007
**28**	Mallotinin	Xu et al., 2007; Trinh et al., 2016	α-glucosidase inhibition	Trinh et al., 2016
			Antioxidant	Xu et al., 2007
**29**	Acetonylgeraniin D	Xu et al., 2007	Antioxidant	Xu et al., 2007
**30**	Corilagin	Zhang et al., 2000b; Xu et al., 2007; Huang et al., 2009; Wu et al., 2012; Trinh et al., 2016	α-glucosidase inhibition	Trinh et al., 2016
			Hepatoprotective	Liu et al., 2017
			Antiinflammatory in cystic fibrosis IB3-1 cells	Gambari et al., 2012
			Mild inhibitory activity against porcine pancreatic amylase	Gunawan-Puteri et al., 2012
			Antioxidant	Xu et al., 2007
			Immunomodulatory	Jantan et al., 2014
			Antiviral	Yeo et al., 2015
**31**	Isostrictinin	Zhang et al., 2000b; Wu et al., 2012		
**32**	Chebulagic acid	Wu et al., 2012		
**33**	Phyllanthusiin C	Huang et al., 2009; Wu et al., 2012		
**34**	Phyllanthusiin B	Wu et al., 2012		
**35**	Phyllanthusiin U	Wu et al., 2012		
**36**	Macatannin B	Gunawan-Puteri et al., 2012	Mild inhibitory activity against porcine pancreatic amylase	Gunawan-Puteri et al., 2012
**37**	Excoecarianin	Cheng et al., 2011	Protected Vero cells from HSV-2 but not HSV-1 infection	Cheng et al., 2011
**38**	Hippomanin A	Yang et al., 2007b	Inhibited HSV-2 but not HSV-1	Yang et al., 2007b
**FLAVONOIDS**				
**39**	Rutin	Yao and Zuo, 1993; Zhang et al., 2000b; Xu et al., 2007; Fang et al., 2008; Thanh et al., 2014	Antioxidant	Xu et al., 2007
				Fang et al., 2008
			Anti *H. pylori*	Lai et al., 2008
**40**	Quercetin 7-methyl ether	Xu et al., 2007	Antioxidant	Xu et al., 2007
**41**	Quercetin 3-O-β-D-glucoside	Xu et al., 2007	Antioxidant	Xu et al., 2007
**42**	Quercitin	Yao and Zuo, 1993; Fang et al., 2008; Wu et al., 2013	Antioxidant, antiinflammatory and anticancer	Fang et al., 2008
			Anti *H. pylori*	Lai et al., 2008
**43**	Rhamnocitrin	Fang et al., 2008	Antioxidant, antiinflammatory and anticancer	Fang et al., 2008
			Anti *H. pylori*	Lai et al., 2008
**44**	Urinariaflavone	Thanh et al., 2014		
**45**	Astragalin or Kaempferol 3-glucoside	Thanh et al., 2014		
**46**	Kaempferol	Yao and Zuo, 1993		
**47**	Quercetin 3-O-α-L-(2,4-di-O-acetyl) rhamnopyranoside-7-O-α-L-rhamnopyranoside	Wu et al., 2013		
**48**	Quercetin 3-O-α-L-(3,4-di-O-acetyl) rhamnopyranoside-7-O-α-L-rhamnopyranoside	Wu et al., 2013		
**49**	Quercetin 3-O-α-L-rhamnopyranoside	Wu et al., 2013		
**50**	4′-Methoxyscutellarein	Tran et al., 2007		
**PHENOLICS**				
**51**	Trimethyl-3,4-dehydrochebulate	Yao and Zuo, 1993; Fang et al., 2008; Hu et al., 2014;	Antioxidant, antiinflammatory and anticancer	Fang et al., 2008
			Anti *H. pylori*	Lai et al., 2008
**52**	Dehydrochebulic acid trimethyl ester	Zhong et al., 1998	Antiviral	Zhong et al., 1998
**53**	Brevifolin	Wu et al., 2012		
**54**	Brevifolincarboxylic acid	Zhang et al., 2000b; Xu et al., 2007; Huang et al., 2009	Antioxidant	Xu et al., 2007
**55**	Methyl brevifolincarboxylate	Yao and Zuo, 1993; Zhong et al., 1998; Fang et al., 2008;	Antioxidant, antiinflammatory and anticancer	Fang et al., 2008
			Anti *H. pylori*	Lai et al., 2008
			Antiplatelet aggregator	Iizuka et al., 2007
			Antiviral	Zhong et al., 1998
**56**	Gallic acid	Yao and Zuo, 1993; Wan et al., 1994; Wei et al., 2005; Xu et al., 2007; Huang et al., 2009; Wu et al., 2012; Hu et al., 2014	Mild inhibitory activity against porcine pancreatic amylase	Gunawan-Puteri et al., 2012
			Antioxidant	Xu et al., 2007
**57**	3,5-Dihydroxy-4-methoxybenzoic acid	Hu et al., 2014		
**58**	Methylgallate	Fang et al., 2008	Antioxidant, antiinflammatory and anticancer	Fang et al., 2008
			Relaxant effect in the guinea pig trachea *in vitro*-: contribution of potassium channels	Paulino et al., 1999
**59**	Ethyl gallate	Santos et al., 1999	*In vivo* antinociceptive	Santos et al., 1999
			Relaxant effect in the guinea pig trachea *in vitro*-: contribution of potassium channels	Paulino et al., 1999
**60**	3, 3′, 4-Tri-O-methylellagic acid	Wan et al., 1994		
**61**	Ferulic acid	Wan et al., 1994; Hu et al., 2014		
**62**	Protocatechuic acid	Xu et al., 2007	Antioxidant	Xu et al., 2007
**63**	2,3,4,5,6-Pentahydroxybenzoic acid	Wei et al., 2005		
**64**	*p*-hydroxybenzaldehyde	Hu et al., 2014		
**65**	Gentisic acid 4-O-β-d-glucopyranoside	Xu et al., 2007	Antioxidant	Xu et al., 2007
**66**	Ellagic acid	Yao and Zuo, 1993; Wan et al., 1994; Shin et al., 2005; Huang et al., 2009; Wu et al., 2012	*In vivo* anti-angiogenic	Huang et al., 2011
			anti-HBV functions	Shin et al., 2005
**67**	Terephthalic acid mono-[2-(4-carboxy-phenoxycarbonyl)-vinyl] ester	Wei et al., 2005;		
**68**	(E)-3- (5′-hydroperoxy-2,2′-dihydroxy[1,1′-biphenyl]-4-yl)-2-propenoic acid	Wei et al., 2005;		
**69**	Syringin	Xu et al., 2007	Antioxidant	Xu et al., 2007
**70**	Phyllanthusiin E	Wu et al., 2012		
**71**	Phyllanthusin F	Zhang et al., 2000a		
**TERPENOIDS**				
***Triterpenoids***				
**72**	β-Amyrin	Agarwal and Tiwari, 1991		
**73**	Glochidiol	Hu et al., 2014		
**74**	Oleanolic acid	Hu et al., 2014		
***Diterpenoids***				
**75**	Cleistanthol	Hu et al., 2014		
**76**	Spruceanol	Hu et al., 2014		
***Sesquiterpenes***				
**77**	Cloven-2β,9α-diol	Hu et al., 2014		
**78**	Dendranthemoside B	Thanh et al., 2014		
***Monoterpenes***				
**79**	(6R)-Menthiafolic acid	Hu et al., 2014		
**80**	Loliolide	Chung et al., 2016	Anti-HCV	Chung et al., 2016
***Steroids***				
**81**	β-sitosterol	Hu et al., 2014		
**82**	(3β,22E)-Stigmasta-5,22-diene-3,25-diol	Hu et al., 2014		
**83**	β-Sitosterol-3-O-β-d-glucopyranoside	Wan et al., 1994; Fang et al., 2008;	Antioxidant, antiinflammatory and anticancer	Fang et al., 2008
			Anti *H. pylori*	Lai et al., 2008
**84**	Stigmasterol	Hu et al., 2014		
**OTHER COMPOUNDS**				
**85**	(+)-Cucurbic acid	Hu et al., 2014		
**86**	(+)-Methyl cucurbate	Hu et al., 2014		
**87**	Methyl (1R,2R,2′Z)-2-(5′-hydroxy-pent-2′-enyl)-3-oxocyclopentaneacetate	Hu et al., 2014		
**88**	(1R,2R)-methyl β-D-glucopyranosyl epituberonate	Thanh et al., 2014		
**89**	Succinic acid	Wan et al., 1994; Wei et al., 2005		
**90**	Phyllanthurinolactone	Ueda et al., 1995	leaf–closing	Ueda et al., 1995
**91**	Triacontanol	Li et al., 1995		
**92**	Lacceroic acid (or dotriacontanoic acid)	Li et al., 1995		
**93**	5-Hydroxymethyl-2-furaldehyde	Hu et al., 2014		

### Anti-diabetic effects of *P. urinaria*

The chronic metabolic disorder, diabetes mellitus is caused by deficiency of insulin secretion and/or decreased response of organs to insulin (Owens et al., 2017). The insulin resistance in type-2 diabetes is normally followed by β-cell dysfunction that causes hyperglycemia (Owens et al., 2017). Commercial drugs are expensive and usually have undesired side effects and toxicity (Owens et al., 2017). Therefore, there is a need to develop an alternative treatment for diabetes. Recent studies focus on the antidiabetic potential of natural products including anti-hypoglycemic or anti-glycation properties and on α-glucosidase inhibition. The enzyme, α-glucosidase cleaves carbohydrates into glucose and elevates the blood glucose level. Therefore, α-glucosidase inhibitors are considered as antidiabetic agents for type-2 diabetes (Dash et al., 2018). The use of natural products as α-glucosidase inhibitors has gained interest because they do not induce toxicity or negative symptoms for the liver, kidney, and gastrointestinal system. Ethanol and water extracts obtained from whole plant of *P. urinaria* inhibit α-glucosidase with IC_50_ values of 39.7 ± 9.7 and 14.6 ± 4.6 μg/mL, respectively (Trinh et al., 2016). A 50% aqueous methanol-soluble extract of the leaves of *P. urinaria* inhibits porcine pancreatic amylase (Gunawan-Puteri et al., 2012). Oral administration of a 50% methanol extract (30 mg/kg) of *P. urinaria* whole plant decreases blood glucose levels (BGL) by 24%, after three h. (Higashino et al., 1992). The *P. urinaria* extract fractionated with *n*-butanol reduced the BGL by 23 and 39% at concentration of 10 and 30 mg/kg, respectively. The 30 mg/kg treatment completely abolished the enhanced BGL (Higashino et al., 1992). The findings emanating from these studies indicated the potential of *P. urinaria* as an antidiabetic agent (Table 1), and this might be explored in the development of new pharmaceuticals. However, the antidiabetic potential of *P. urinaria* needs further study including protection of pancreatic β-cells against oxidative damage and insulin secretion and postprandial blood glucose levels in models *in vitro* and *in vivo*.

### Antimicrobial activity of *P. urinaria*

Antimicrobial activity of *P. urinaria* is indicated in Table 1. It known that *Helicobacter pylori* is resistant to most antibiotics, but *P. urinaria* preparations have antimicrobial activity against this bacterium. Chloroform and methanol extracts of *P. urinaria* whole plant have superior anti-*H. pylori* activity compared with its pure compounds (Lai et al., 2008). The chloroform extract potently inhibits *H. pylori* adhesion and invasion of gastric epithelial AGS cells, whereas the methanol extract has a moderate effect. The chloroform extract attenuates *H. pylori*-induced NF-κB activation with subsequent release of IL-8 (Lai et al., 2008). The anti-plasmodial activity *in vitro* of aqueous, methanol, and dichloromethane extracts of *P. urinaria* whole plant against a chloroquine-resistant *Plasmodium falciparum* strain (W2) indicates that the methanolic extract of *P. urinaria* is as active as the dichloromethane extract (IC_50_ values of ≤ 4 μg/mL; Hout et al., 2006). The methanolic extract of *P. urinaria* whole plant also has potent anti-malarial activity toward chloroquine-sensitive (CQS) strains of *P. falciparum* with an IC_50_ = 4.1 μg/mL (Haslinda et al., 2015). The mechanism behind the antimicrobial action of *P. urinaria* extracts is associated with the presence of metabolites including phyllanthin, phyltetralin, rutin, quercetin, trimethyl-3,4-dehydrochebulate and methyl brevifolincarboxylate (Table 2). These compounds present in *P. urinaria* extracts may interact with the proteins present in the microbial cell membrane to form stable water-soluble complexes, resulting in microbial cell death.

### Cardioprotective effects of *P. urinaria*

In recent years, there is interest in naturally occurring cardioprotective agents that may lack side effects. Herbal products are widely used among patients with cardiovascular (CV) diseases, and patients often combine herbal products with CV medications. Extracts of *P. urinaria* have cardio-protective effects *in vitro* in streptozotocin-induced diabetic rats (Table 1). The ethanolic extract of *P. urinaria* whole plant has antioxidant and cardioprotective effects against doxorubicin toxicity in H9C2 cardiac myoblasts (Chularojmontri et al., 2009). The ethanolic extract from the aerial parts of *P. urinaria* increase the activity of catalase/superoxide dismutase, increase total glutathione concentration and inhibit lipid peroxidation. The extract induces apoptosis in H9c2 cells through the NF-κB and caspase-3 activation signaling pathway (Chularojmontri et al., 2005, 2009). These studies indicate that crude extracts of *P. urinaria* have cardioprotective potential and might lead to promising agents for therapeutic development to treat cardiac complications.

### Other activities of *P. urinaria* extracts

Ethanol extracts of *P. urinaria* whole plant stimulate antiarthritic activity *in vitro* (Buddhachat et al., 2017). The methanol extract obtained from whole plant of *P. urinaria* increases phagocytosis of human phagocytes (Yuandani et al., 2013). Extracts of *P. urinaria* promote N-cadherin expression *in vivo* in the testicular tissues disrupted by nitrogen mustard (Zhang et al., 2008). The hydro-alcoholic extract of *P. urinaria* whole plant prevents 7,12-dimethylbenz(a)anthracene (DMBA)-induced skin papillomagenesis *in vivo* (Bharali et al., 2003). A fraction containing 60% corilagin obtained from whole plant of *P. urinaria* has antithrombosis activity through inhibition of platelet-neutrophil adhesion (Shen et al., 2004).

## Phytochemical constituents of *P. urinaria*

**Figure 1:**
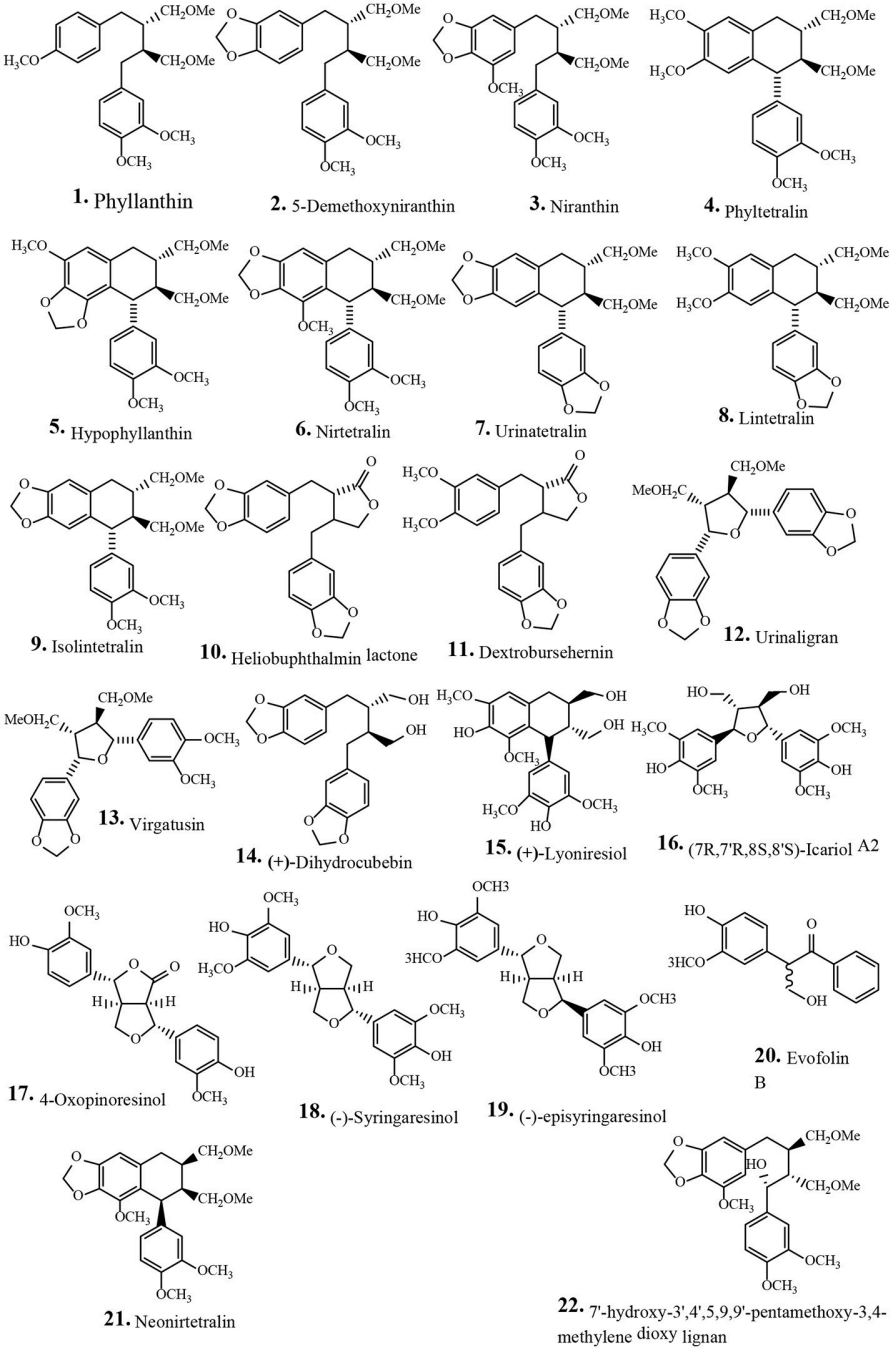
Chemical structures of isolated lignans from *Phyllanthus urinaria*.

**Figure 2:**
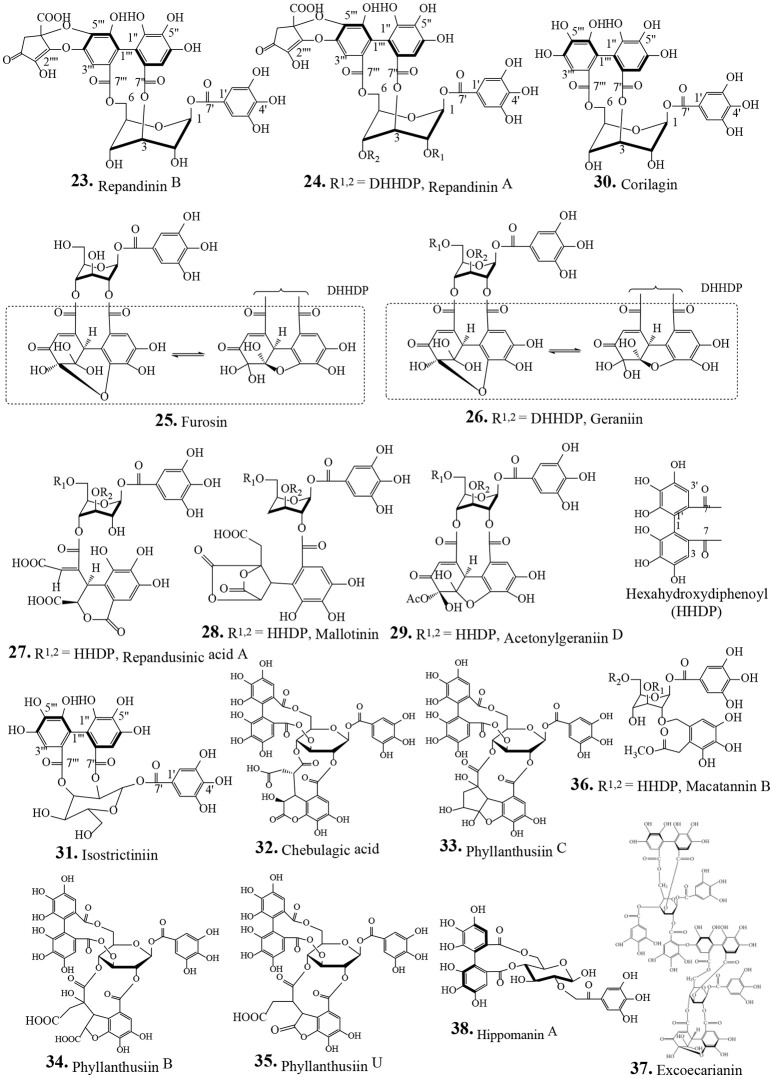
Chemical structures of isolated tannins from *Phyllanthus urinaria*.

**Figure 3:**
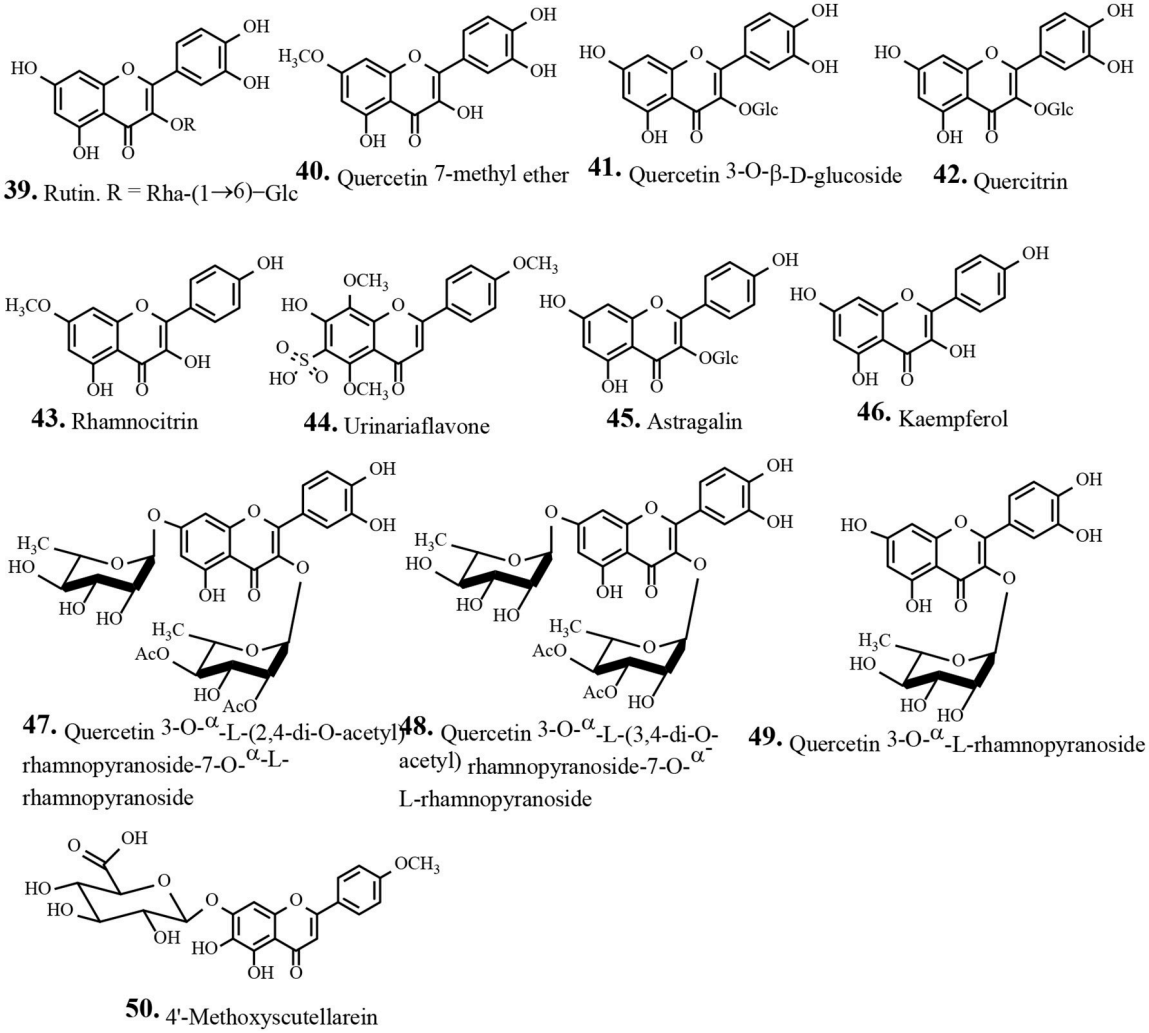
Chemical structures of isolated flavonoids from *Phyllanthus urinaria*.

**Figure 4:**
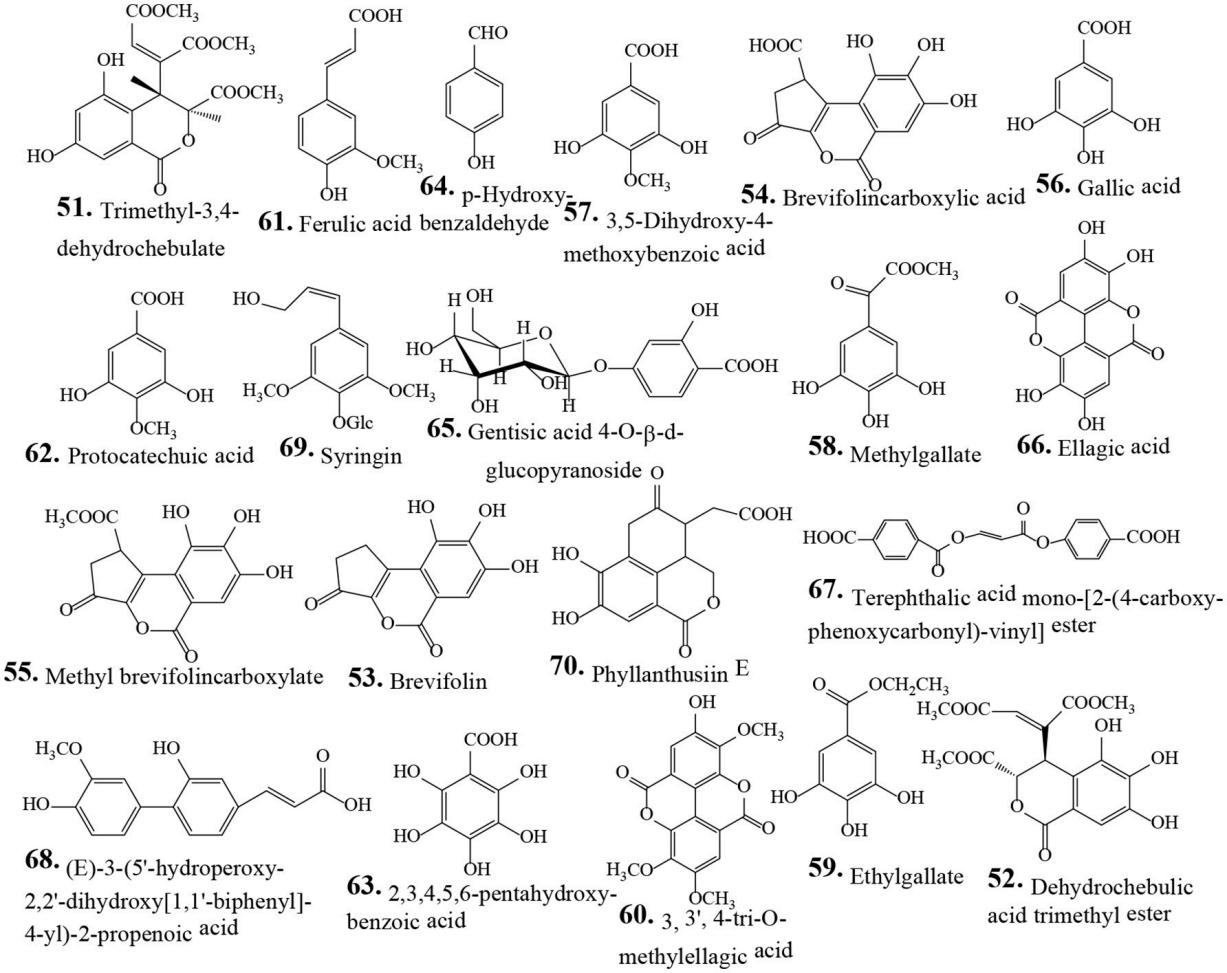
Chemical structures of isolated phenolic compounds from *Phyllanthus urinaria*.

**Figure 5:**
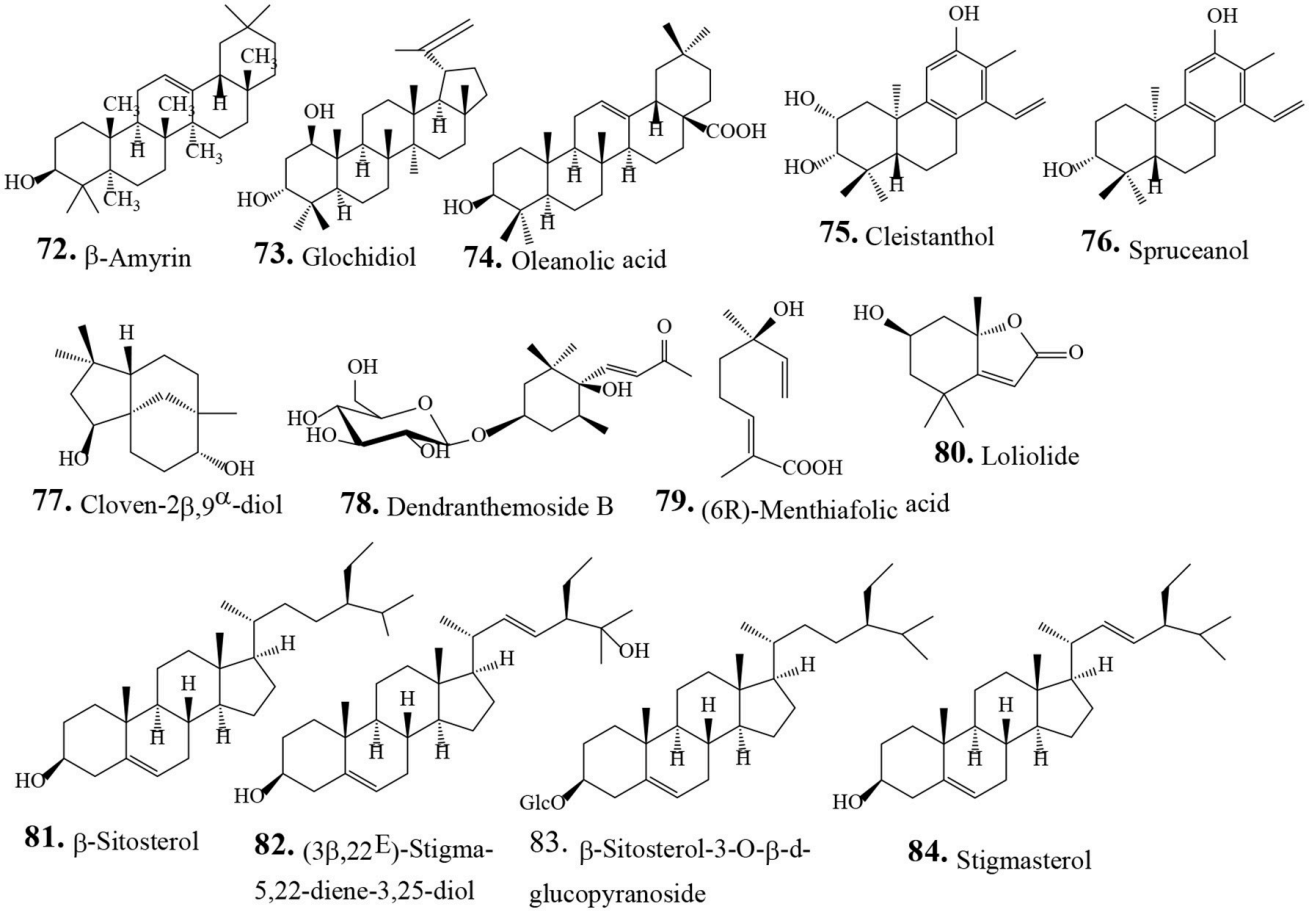
Chemical structures of isolated terpenoids from *Phyllanthus urinaria*.

**Figure 6:**
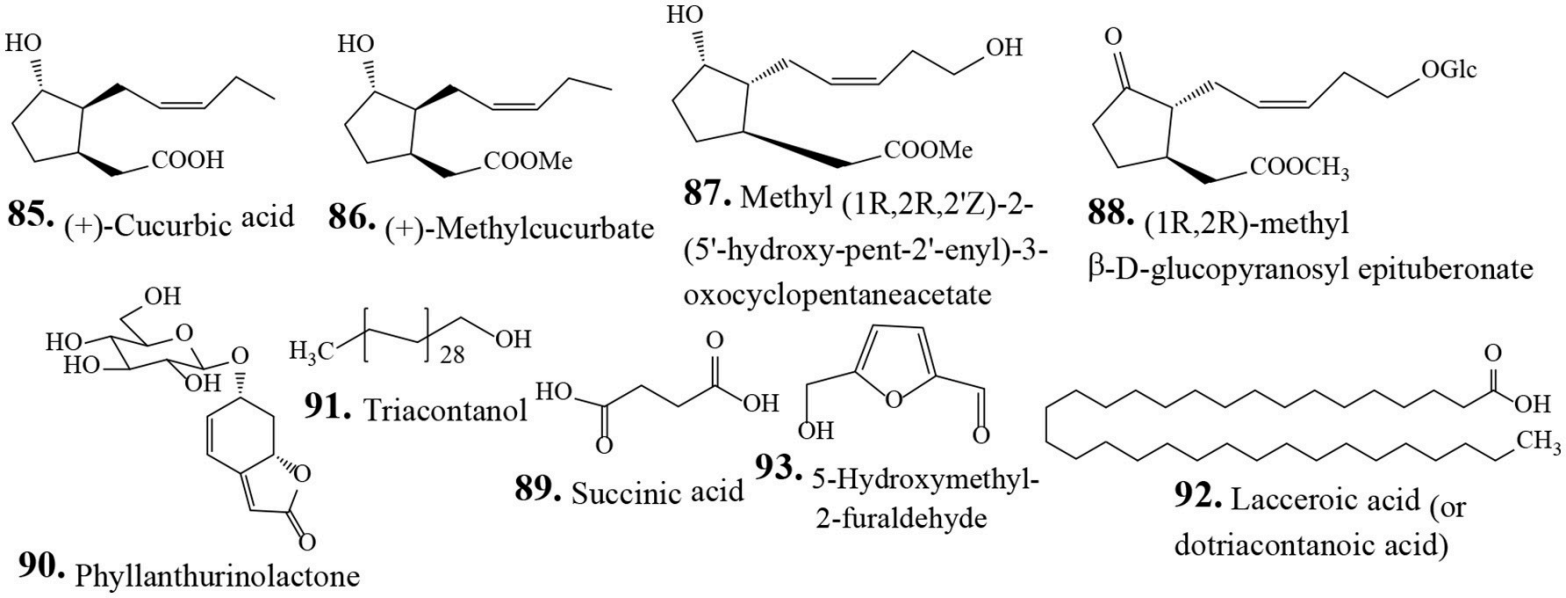
Chemical structures of isolated other compounds from *Phyllanthus urinaria*.

Traditionally human populations consume herbs and their extracts. Many modern medicines use standardized plant extracts as active constituents. Various phytochemical groups have been isolated and identified from *P. urinaria* by chromatographic techniques. These constituents include lignans, tannins, flavonoids, phenolic acids, terpenoids, and other compounds (Table 2). To date, 93 compounds have been identified and structurally elucidated from the extracts of *P. urinaria* including 22 lignans, 16 tannins, 12 flavonoids, 21 phenolics, 13 terpenoids, and other secondary metabolites (Table 2). Typical structures of isolated constituents from *P. urinaria* are shown in Figures 1–6. The chemical profiles of *P. urinaria* may vary with the geographical production region, plant organs used and extraction procedure. Lignans and tannins exhibit various activities and are considered the major biological active compounds of *P. urinaria* (Satyan et al., 1995; Zhong et al., 1998; Liu et al., 1999; Giridharan et al., 2002; Yang et al., 2007a,b; Fang et al., 2008; Cheng et al., 2011; Huang et al., 2011). Corilagin, geraniin, and gallic acid are the three most prevalent compounds in *P. urinaria*, and pharmacological researches mainly focus on phyllanthin, hypophyllanthin, corilagin, geraniin, brevifolin and its derivatives, and rutin. The list of compound names and their biological activities are presented in Table 2.

### Lignans

Lignans are phenyl propanoid inter-unit linkage metabolites, which play an important role in plant defense systems. These compounds consist of different groups such as dibenzylbutane, arylnaphthalene, dibenzylbutyrolactone, aryltetralin, tetrahydrofuran, and furofuran. Lignans have a wide range of biological activities including antioxidant, anticarcinogenic, estrogenic, antiviral, and antihypertensive properties (Xu et al., 2018). The pharmaceutical industries use an aryltetralin lignan, podophyllotoxin as a precursor for the synthesis of the anticancer drug etoposide. Lignans affect adverse estrogen activities and attenuate hormone-associated cancers including breast, ovarian, and uterine cancers (Xu et al., 2018). Thirteen lignans have been isolated from the aerial and root parts of *P. urinaria* including four novel compounds, namely 5-demethoxyniranthin (**2**), urinatetralin (**7**), dextrobursehernin (**11**), and urinaligran (**12**) (Chang et al., 2003; Wang and Lee, 2005). Hu et al. (2014) reported the isolation of seven lignans from *P. urinaria* whole plants including three bistetrahydrofuran lignans, 4-oxopinoresinol (**17**), (-)-syringaresinol (**18**) and (-)-episyringaresinol (**19**) (Table 2, Figure 1). Some of the lignans isolated from *P. urinaria* extracts have interesting biological activities (Table 2). For example, phyllanthin (**1**), is traditionally applied in the treatment of many liver diseases and has antioxidant, anti-inflammatory, immunomodulatory, and hepatoprotective activities (Table 2). In particular, phyllanthin (**1**) attenuates the CCl_4_ and galactosamine induced cytotoxicity in rat hepatocytes (Krithika et al., 2009). Additionally, phyllanthin has antioxidant activities including inhibition of superoxide dismutase (SOD) and glutathione reductase enzymes and attenuates ethanol-induced oxidative damage in rat hepatocytes (Chirdchupunseree and Pramyothin, 2010). Neonirtetralin (**21**) has cytotoxic effects in CHO and J774 cell lines with IC_50_ values of 8.07 and 6.00 μM, respectively (Thanh et al., 2014). Moderate cytotoxic activity is observed for hypophyllanthin (**5**) and heliobuphthalmin lactone (**10**) against CHO and J774 cell lines with IC_50_ values ranging from 15.82–41.30 μM (Thanh et al., 2014). 7'-Hydroxy-3',4',5,9,9'-pentamethoxy-3,4-methylenedioxy lignan (**22**) has anti-proliferative properties in Hep2, EL-1 monocytes, HeLa and MCP7 cells, and induces apoptosis through inhibited telomerase activity and activation of c-myc and caspase 3 and 8 (Giridharan et al., 2002). Compounds **1** and **5** have vasorelaxation effects *in vitro* in rat aorta (Inchoo et al., 2011). These reports indicate that isolated lignans from *P. urinaria* have potential biological activities including anticancer and hepatoprotective effects (Table 2).

### Tannins

Tannins are water-soluble polyphenolic biomolecules present in many plant foods. Tannins consist of two groups; one is the hydrolysable tannins containing gallic and/or ellagic acids with sugar moieties; the second one is condensed tannins (proanthocyanidins) which contain catechin and epicatechin oligomers. Tannins interact with one or more protein molecules to form water insoluble complexes. Tannins have various beneficial biological activities including anticancer, cardio-protective, antimicrobial, antioxidant and free radical scavenging activities (Smeriglio et al., 2017). All of the *P. urinaria* tannins (**23**–**38**, Table 2) are hydrolysable tannins, characterized by the presence of one or more galloyl, hexahydroxydiphenol (HHDP) or HHDP metabolites attached to a glucopyranose core unit. Seven ellagitannins have been isolated from an aqueous acetone extract of the whole plant *P. urinaria* (**23**, **25**–**30**) (Xu et al., 2007). From the hot water extract of *P. urinaria*, the tannin compounds, geraniin (**26**), corilagin (**30**), isostrictinin (**31**) chebulagic acid (**32**), phyllanthusiin C (**33**), phyllanthusiin B (**34**) and phyllanthusiin U (**35**) are identified (Wu et al., 2012). The compounds **30** and phyllanthusiin C (**33**) are identified as markers of *P. urinaria* (Huang et al., 2009). Compounds **26** and **30** are major tannins obtained from *P. urinaria*; they have potent DPPH-radical-scavenging and mushroom-tyrosinase-inhibitory activities (Xu et al., 2007). Compound **30** has antiviral activity evidenced by reduced coxsackievirus A16 (CA16), and human enterovirus 71 (EV71)-induced cytotoxicity in Vero cells with IC_50_ = 5.6 and 32.33 μg/mL, respectively (Yeo et al., 2015). Many of the tannins exhibit multiple activities such as antioxidant, antitumor, and hepatoprotective activities (Table 2). It is known that HSV, both type 1 (HSV-1) and type 2 (HSV-2), can lead to the development of genital herpes, particularly HSV-2. Hippomanin A (**38**) and **30** isolated from the acetone extract of *P. urinaria* act differently in suppressing HSV infection. The isolate **30** did not affect HSV-1 or HSV-2 infection, but compound **38** prevented HSV-2 infection with no effect on HSV-1 replication (Yang et al., 2007b). Corilagin (**30**) has anti-inflammatory activity in cystic fibrosis bronchial IB3-1 cells involving inhibition of NF-κB/DNA interactions, IL-8 gene expression, and MCP-1 and RANTES secretion (Gambari et al., 2012). Tannin **26** has antioxidant and anti-semicarbazide-sensitive amine oxidase activities *in vitro* and anti-hypertensive activities *in vivo* (Lin et al., 2008). Compound **30** protects against hemorrhagic shock-induced liver injury through the Akt-dependent pathway (Liu et al., 2017). These results indicate that tannins isolated from *P. urinaria* have important biological functions and deserve further study (Table 2).

### Flavonoids

Flavonoids, are a group of natural substances consisting of two aromatic rings joined by a three carbon-oxygenated heterocycle. These are the most numerous group of polyphenolic phytonutrients (plant chemicals) and are found in most fruits and vegetables. Flavonoids have various pharmacological activities including anticancer, anti-inflammatory, antioxidant, anti-diabetic, and antiviral activites through various cell-signaling pathways (Mozaffarian and Wu, 2018). Most of the flavonoids reported from *P. urinaria* are in the flavonol and glycoside form (Nara et al., 1977) (Table 2). From the ethanolic extract of *P. urinaria*, two new acetylated flavonoid glycosides **47**, **48**, along with the known isolates, quercetin (**42**) and quercetin 3-O-α-L-rhamnopyranoside (**49**) have been isolated (Wu et al., 2013). A new flavone sulfonic acid, urinariaflavone (**44**) was isolated from the methanolic extract of *P. urinaria* (Thanh et al., 2014). The isolated flavonoids from *P. urinaria* showed antioxidant, anti-inflammatory, anticancer, and anti-*H. pylori* etc., activities (Table 2).

### Phenolics

Phenolic compounds are the major group of phytochemicals that include at least one aromatic ring, with one or more hydroxyl groups attached. Phytochemical investigation of ethanolic extract from whole plants of *P. urinaria* resulted in the isolation of nine compounds including trimethyl-3,4-dehydrochebulate (**51**), methylgallate (**58**), and methyl brevifolincarboxylate (**55**) (Fang et al., 2008). The isolates **51**, **55**, and **58** have DPPH radical scavenging activity with IC_50_ values of 9.4, 8.9, and 9.8 μM, respectively. These isolates dose-dependently inhibit the enhanced production of NO radicals, and TNF-α and IL-6 in LPS/IFN-γ-activated macrophages (Fang et al., 2008). Five carboxylic acids including two new ones, terephthalic acid mono-[2-(4-carboxy-phenoxycarbonyl)-vinyl] ester (**67**), and (E)-3-(5'-hydroperoxy-2,2'-dihydroxy[1,1'-biphenyl]-4-yl)-2-propenoic acid (**68**) were isolated from the *n*-butanol fraction from methanolic extract obtained from whole plants of *P. urinaria* (Wei et al., 2005). Five major compounds including gallic acid (**56**), brevifolin carboxylic acid (**54**), and ellagic acid (**66**) were identified as markers of *P. urinaria* (Huang et al., 2009). From the hot water extract of *P. urinaria*, the phenolic compounds brevifolin (**53**), **54**, **56**, **66**, and Phyllanthusiin E (**70**) are also identified (Wu et al., 2012). The polyphenolic compound, phyllanthusin F (**71**) was isolated from ethanolic extract obtained from whole plants of *P. urinaria* (Zhang et al., 2000a). Compound **66** has significant antihepatotoxic activity. The antiangiogenic activity of **66** was observed in HUVEC cells by its inhibitory effect on cell migration and MMP-2 secretion (Huang et al., 2011). From the aerial parts of *P. urinaria*, compound **59** (gallic acid ethyl ester) was isolated and has antinociceptive activity *in vivo* (Santos et al., 1999). Compound **66** (ellagic acid) has no noticeable effect on HBV replication and its polymerase activity or on HBsAg secretion. However, it potently inhibits HBeAg secretion in HepG2 2.2.15 cells with an IC_50_ of 0.07 μg/mL (Shin et al., 2005). These results indicate that *P. urinaria* is a source for biologically important phenolic compounds including trimethyl-3,4-dehydrochebulate (**51**), brevifolin (**53**) and its derivatives (**54**, **55**), gallic acid (**56**), and its derivatives (**57**–**60**) and ellagic acid (**66**).

### Terpenoids

Terpenoids (or isoprenoids) are compounds derived from one or more five-carbon isoprene units. These compounds represent the most diverse class of beneficial phytochemicals with anticancer, anti-cardiovascular, anti-Alzheimers, and anti-malarial activities. The terpenoids such as taxol, artemisinin, and ginkgolides have therapeutic effects on a variety of diseases (Cho et al., 2017). A number of terpenoids (13 compounds) including three triterpenoids (**72**–**74**), two diterpenoids (**75** and **76**), two sesquiterpenes (**77** and **78**), two monoterpenes (**79** and **80**), and four sterols (**81**–**84**) have been isolated from the extracts of *P. urinaria* (Table 2). Fractionation of the acetone extract from *P. urinaria* resulted in the isolation of a monoterpenoid lactone, loliolide (**80**) that has anti-HCV activity through inactivation of virus particles, revocation of HCV attachment and reduced viral fusion (Chung et al., 2016). The pentacyclic oleanane-type triterpenoid β-amyrin (**72**) has anti-inflammatory, anti-nociceptive, antimicrobial, and anti-apoptotic activities (Askari et al., 2018). Oleanolic acid (**74**) and its derivatives have therapeutic potential against various types of cancers *in vitro* and *in vivo* (Ayeleso et al., 2017).

### Other compounds

Chemical examination of a 95% ethanol extract obtained from whole *P. urinaria* plants results in the isolation of twenty-three compounds including three jasmonate derivatives, (+)-cucurbic acid (**85**), (+)-methyl cucurbate (**86**), methyl (1R,2R,2′Z)-2-(5′-hydroxy-pent-2′-enyl)-3-oxocyclopentaneacetate (**87**), and 5-hydroxymethyl-2-furaldehyde (**93**) (Hu et al., 2014). The methanolic extract obtained from the whole *P. urinaria* plant results in the isolation of phyllanthurinolactone (**90**) that stimulates leaf closing of *P. urinaria* in the daytime, without affecting other nyctinastic plants (Ueda et al., 1995).

## Clinical trials of *P. urinaria* preparations

It known that clinical trials are required for any new compound to enter into the market. Table 3 summarizes the important clinical trials of *P. urinaria*. In China, 140 chronic hepatitis B patients treated for two years have a recovery rate expressed as the index of HBV-DNA and HBeAg of 88.2% and 52.5%, respectively. Once the treatment is stopped, the recurrence rate is 10.4–13.4% respectively (Cheng et al., 2009). Tong et al. (2014) reports that compound in capsule of *P. urinaria* L. suppresses development of hepatocellular carcinoma (HCC) through an improved immune system, reversion of liver fibrosis, blockage of the induced hepatocarcinoma cell cycle and inhibition of angiogenesis. The HBV-DNA levels decrease ≥2 log in 22.2% (10/45) of patients in the treatment group compared with the control group at 5.0% (2/40). The number of antibodies that test positive in the treated group is lower (1.08 ± 1.01) after the treatment period of 24 months compared with the control group (2.11 ± 1.12) (Tong et al., 2014). The anti-URG11 (33/52) and anti-URG19 (31/52) in both treated and control groups are over 60% at base line. After the treatment period of two years, in the treated group the levels of anti-URG11 and anti-URG19 decreased to 48.1% (25/52) and 46.2% (24/52), whereas in the control group the anti-URG11 and anti-URG19 levels were at relatively higher values of 68.0% (34/50), and 66.0% (33/50), respectively. Wang et al. (1995) report on 35 patients receiving a *P. urinaria* extract and thirty-five control patients; there was no detectable hepatitis B e-antigen in patient's serum after treatment with *P. urinaria*. No patient changed status with respect to hepatitis B s-antigen (Wang et al., 1995). In contrast to the above results of anti-HBV effects of *P. urinaria*, an another study indicates that *P. urinaria* treatment for 6 months has no effect on HBV patients including no variation in log10 [HBV DNA] reduction using *P. urinaria* at 1 g (0.18 ± 1.42), 2 g (0.33 ± 1.08), or 3 g (0.85 ± 1.30) compared to a placebo (0.28 ± 0.85). Also there was no difference in the HBeAg conversion and ALT normalization of treated compared to control groups (Chan et al., 2003). Wong et al. (2013) using a tablet containing 400 mg of *P. urinaria* for 24 weeks find no improvement in non-alcoholic steatohepatitis (NASH) and non-alcoholic fatty liver disease (NAFLD). Histologically, there is a minor reduction in steatosis and hepatocyte ballooning in the treated group, however, it is not significant. Perhaps, *P. urinaria* might not be a suitable agent to treat NASH (Wong et al., 2013).

**Table 3:** Reported Clinical trials of *Phyllanthus urinaria*.

**Sample**	**Result**	**Reference**
Compound *P. urinaria* L (CPUL)	CPUL prevented or delayed in the development of HBV-associated cirrhosis to HCC through improved immune system, revert liver fibrosis, induced hepatocarcinoma cell cycle block and inhibited angiogenesis.	Tong et al., 2014
400 mg of *P. urinaria* tablet	*Phyllanthus* is not superior to placebo in improving NAFLD activity score in NASH patients	Wong et al., 2013
*Phyllanthus* Pill	After treatment with *P. urinaria* capsule for 3 months or 2 years, the recovery rate in the index of HBV-DNA and HBeAg was 88.2% and 52.5%, respectively.	Cheng et al., 2009
*P. urinaria* extract	Received *P. urinaria* 1, 2 and 3g three times daily for 6 months, there was no difference in log10 [HBV DNA] reduction, HBeAg seroconversion and ALT normalization, suggested *P. urinaria* had no demonstrable anti-viral effect in chronic hepatitis B	Chan et al., 2003
*P. urinaria* extract	Patients received *P. urinaria* extract lose detectable hepatitis B e-antigen from their serum and likely to seroconvert hepatitis B e-antibody status from negative to positive	Wang et al., 1995

### Toxicology

Little data is available regarding the toxicity profiles of *P. urinaria* preparations. Chan et al. (2003) demonstrates that *P. urinaria* is well tolerated for 6 months by both male and female patients aged between 18 and 65 with positive hepatitis B surface antigen (HBsAg). There is no difference in toxicological measurements between treated and control groups; in both groups some subjects experienced mild negative effects.

## Future prospects

This review summarizes information regarding the traditional uses of phytochemicals, pharmacological activities of crude extracts as well as pure compounds, analysis of active compounds, and clinical trials related to *P. urinaria*. There is evidence that the crude extracts and pure compounds found within *P. urinaria* have anticancer, hepatoprotective, antimicrobial, antidiabetic, and cardioprotective activities through various signaling pathways. Although the chemical structure and its biological potential of some of the constituents are known, generally, the mechanisms of action need to be investigated for further development into therapeutics.

Systematic efficacy studies are necessary to examine standardized extracts of *P. urinaria* and to identify the bioactive molecules responsible for the pharmacological activities. If possible, specific targets (i.e., receptors) need to be identified. The reported clinical data for *P. urinaria* against HBV is limited and consequently limits the use of herbal medicines to treat chronic liver disease. The compounds brevifolin and its derivatives, corilagin, ellagic acid, gallic acid, geraniin, loliolide, phyllanthin may be drug candidates for treating liver diseases because of their potent antiviral activites including anti-hepatitis activity. The high concentration of these compounds in *P. urinaria* suggests their use and indicates that studies are needed to assess the absorption, distribution, metabolism, and excretion of candidate compounds. Mechanism of action studies on the liver protecting effect of *P. urinaria* preparations and purified compounds when combined with conventional medicines, are also expected to lead the way in the discovery of new agents with improved pharmacological properties.

The herbal medicines cultivated in different geographical regions differ in their composition as well as their therapeutic effects demanding quality control of *P. urinaria* preparations and toxicological studies. Toxicological studies need to address the mycotoxin, heavy metal, and pesticide concentrations as well as the general toxicity of *P. urinaria* extracts and purified compounds. Attempts need to be made to gain regulatory approval of *P. urinaria* preparations as nutraceuticals or medicinal drugs.

## Author contributions

MG wrote the manuscript. S-TD edited the manuscript. Both authors have seen and agreed on the finally submitted version of the manuscript.

### Conflict of interest statement

The authors declare that the research was conducted in the absence of any commercial or financial relationships that could be construed as a potential conflict of interest. The reviewer SC and handling Editor declared their shared affiliation.
